# Structure of the *Dictyostelium* Myosin-II Heavy Chain Kinase A (MHCK-A) α-kinase domain apoenzyme reveals a novel autoinhibited conformation

**DOI:** 10.1038/srep26634

**Published:** 2016-05-23

**Authors:** Qilu Ye, Yidai Yang, Laura van Staalduinen, Scott William Crawley, Linda Liu, Stephanie Brennan, Graham P. Côté, Zongchao Jia

**Affiliations:** 1 Department of Biomedical and Molecular Sciences, Queen’s University, Kingston, ON, K7L 3N6, Canada

## Abstract

The α-kinases are a family of a typical protein kinases present in organisms ranging from protozoa to mammals. Here we report an autoinhibited conformation for the α-kinase domain of Dictyostelium myosin-II heavy chain kinase A (MHCK-A) in which nucleotide binding to the catalytic cleft, located at the interface between an N-terminal and C-terminal lobe, is sterically blocked by the side chain of a conserved arginine residue (Arg592). Previous α-kinase structures have shown that an invariant catalytic aspartic acid residue (Asp766) is phosphorylated. Unexpectedly, in the autoinhibited conformation the phosphoryl group is transferred to the adjacent Asp663, creating an interaction network that stabilizes the autoinhibited state. The results suggest that Asp766 phosphorylation may play both catalytic and regulatory roles. The autoinhibited structure also provides the first view of a phosphothreonine residue docked into the phospho-specific allosteric binding site (Pi-pocket) in the C-lobe of the α-kinase domain.

*Dictyostelium* myosin-II heavy chain kinase A (MHCK-A) phosphorylates the α-helical coiled-coil tail of myosin-II, causing contractile bipolar filaments of myosin-II to disassemble into inactive monomers^1^,^2^,^3^,^4^. By inhibiting myosin-II, MHCK-A plays a central role in the regulation of cellular processes such as cytokinesis, chemotaxis and the maintenance of cortical tension^5^,^6^,^7^,^8^,^9^,^10^,^11^. MHCK-A is a member of the atypical α-kinase family of serine/threonine protein kinases^12^,^13^. The α-kinase domain of MHCK-A is flanked by an N-terminal coiled-coil region and a C-terminal WD-repeat domain (Fig. 1)^14^,^15^. The coiled-coil region self-assembles into trimers or tetramers, so that when examined by rotary shadowing electron microscopy MHCK-A appears as an elongated protein with multiple globular domains clustered together at one end of a 50 nm-long rod-like segment^16^. The coiled-coil region targets MHCK-A to actin-rich regions of the cell, such as the leading edge of pseudopods^17^,^18^. The WD-repeat domain binds myosin-II filaments and is required for MHCK-A to effectively phosphorylate myosin-II in cells^19^.

Members of the α-kinase family exist in organisms ranging from single-celled protozoa to mammals. Well-characterized members of the α-kinase family include eukaryotic elongation factor 2 kinase (eEF2K), which inhibits ribosomal protein translation by inactivating eEF2^20^,^21^, and transient receptor potential melastatin 7 (TRPM7), which also functions as a nonselective cation channel^22^. The α-kinase domain of TRPM7 phosphorylates the tail of myosin-II to inhibit filament assembly^23^,^24^ and, when cleaved from TRPM7, can enter the nucleus to phosphorylate histones^25^.

The α-kinases share little primary sequence similarity with other protein kinases. Nevertheless, X-ray crystal structures of MHCK-A and TRPM7 show that the α-kinase fold and active site resemble those of other protein kinases^26^,^27^. The α-kinase domain is bi-lobed, with the active site located in a large cleft at the junction of the two lobes. The α-kinase N-lobe is similar to the N-lobe of other protein kinases but the C-lobe has a distinctive structure and requires a tightly bound zinc atom for stability. A unique feature of the α-kinase C-lobe is the N/D-loop, which forms the wall facing the triphosphate half of ATP in the inter-lobe cleft and is involved in peptide substrate recognition^28^. The C-lobes of MHCK-A and eEF2K contain an allosteric site, the Pi pocket, which specifically binds phosphopeptides^28^,^29^,^30^. An intramolecular ligand for the Pi-pocket can be generated by the autophosphorylation of a conserved threonine residue (Thr825 in MHCK-A; Thr348 in eEF2K) in the unstructured linker C-terminal to the α-kinase domain. Autophosphorylation of Thr825/Thr348 is critical for the activation of MHCK-A and eEF2K^28^,^29^,^30^.

The α-kinase active site contains a number of catalytic residues conserved in all members of the protein kinase-like superfamily^31^,^32^. However studies on A-CAT, the α-kinase domain of MHCK-A, reveals that its catalytic properties differ in significant ways from those of other protein kinases. A-CAT can utilize both ATP and ADP to phosphorylate peptides and proteins and is able to remove all three phosphoryl groups from ATP to generate adenosine. These unusual catalytic activities can be attributed to distinctive active site features, such as the presence of an invariant basic residue (Arg592; numbering for MHCK-A) in the phosphate-binding loop (P-loop) in the N-lobe. Electrostatic repulsion with Arg592 positions the side chain of the invariant Lys645 residue (equivalent to Lys72 in the cAMP-dependent kinase (PKA)) between the adenine base and α-phosphoryl group, where it may help promote removal of the α- and β-phosphoryl groups. In several crystal structures of A-CAT a catalytically essential aspartic acid residue in the C-lobe (Asp766) is phosphorylated^27^. Asp766 is phosphorylated in structures of A-CAT bound to ADP, AMP or adenosine, suggesting that it is able to accept all three phosphoryl groups. Conventional protein kinases, such as PKA, do not form a phosphoenzyme intermediate^33^. However, the residue equivalent to Asp766 is also phosphorylated in the atypical RIO kinases, where it drives a conformational change that regulates binding interactions^34^,^35^,^36^. Thus, it is possible that Asp766 phosphorylation serves a dual function, both as an intermediate in the catalytic mechanism and as a regulatory switch.

In this study we sought to gain further insights into the conformational dynamics of the α-kinase domain. We hypothesized that nucleotide binding might be required for the α-kinase domain to achieve a catalytically competent conformation and so have solved the crystal structure of A-CAT in the absence of nucleotide (Apo-A-CAT). The structure of Apo-A-CAT provides the first view of a phosphopeptide bound to the Pi-pocket and reveals a novel open, autoinhibited conformation for the catalytic cleft. Surprisingly, Asp663, and not Asp766, is phosphorylated in Apo-A-CAT. To further investigate the function of Asp663 we also solved the crystal structure of an A-CAT-D663A mutant.

## Results

### Organization of molecules in the Apo-A-CAT crystal structure

**Figure 1:**
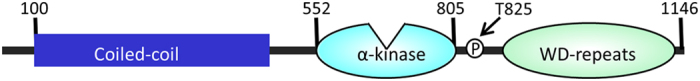
Schematic diagram showing the domain organization of MHCK-A. MHCK-A consists of an N-terminal coiled-coil domain (left), a central α-kinase domain and a C-terminal WD repeat domain (right). The Thr825 site of autophosphorylation required for activity is indicated by a P.

**Figure 2:**
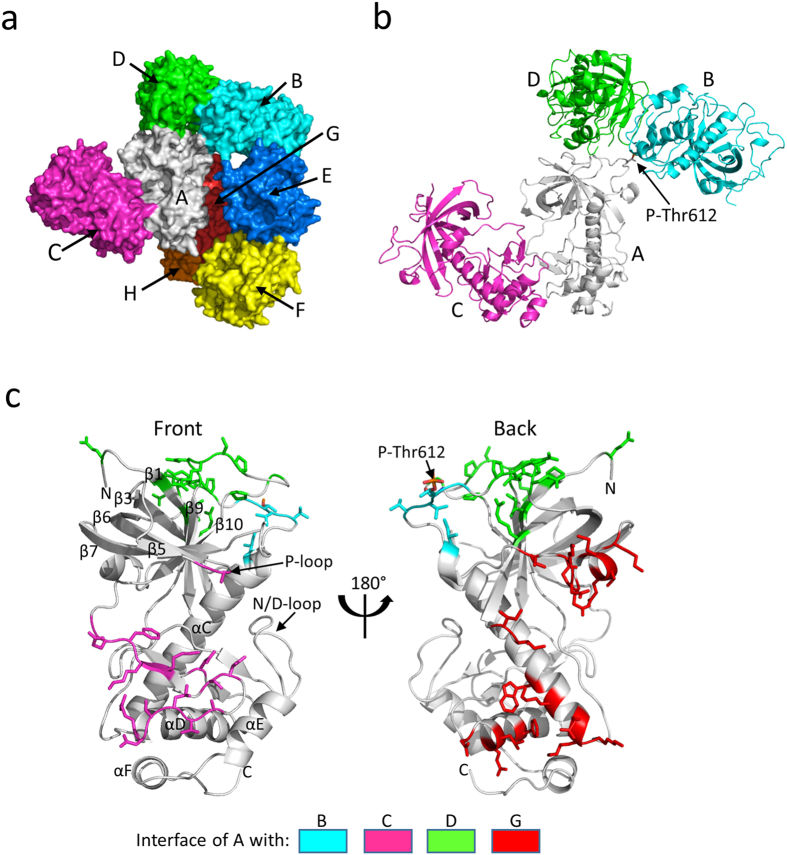
Organization of molecules in the Apo-A-CAT asymmetric unit. (**a**) Surface view of the Apo-A-CAT asymmetric unit with the eight monomers labeled A-H. (**b**) Cartoon representation of the A-D tetramer in the same orientation and coloring as in panel A. The phosphothreonine (P-Thr612) in B that binds to the Pi pocket in A is shown in stick representation. (**c**) Cartoon representation showing the front and back of molecule A. Residues involved in interfaces with B, C, D and G are shown as sticks and colored as indicated in the legend. Labels indicate the N- and C-termini (N and C), α-helices, β strands, N/D-loop, P-loop and P-Thr-612.

A-CAT encompasses the α-kinase domain and Thr825 autophosphorylation site of MHCK-A (Fig. 1). We purified and crystallized A-CAT in the absence of nucleotide, and were able to obtain a crystal structure for the apoenzyme (Apo-A-CAT) at 2.9 Å resolution. Data collection, processing, and refinement statistics are provided in Table 1. The asymmetric unit contained eight monomers of Apo-A-CAT (labeled A to H) organized into two nearly identical tetramers (A-D and E-H) (Fig. 2a,b). The A-D tetramer is chosen as a representative for examination of the interaction between molecules. In the A-D tetramer, the core A molecule forms interfaces with molecules B, C and D as well as with the equivalent molecule (G) in the other tetramer. There is also an interface between molecules B and D, which is similar as the one between molecules A and C. Detailed information for each interface is provided in Table S1.

**Figure 3:**
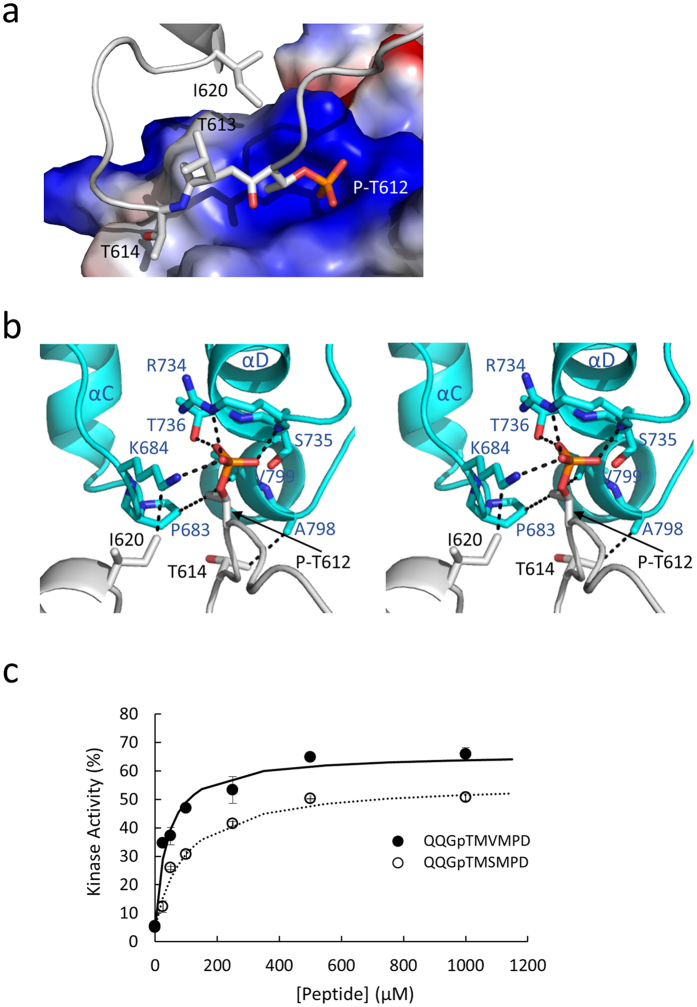
Binding of P-Thr612 to the Pi-pocket. (**a**) The Pi-pocket region in molecule B is shown as a surface representation and is colored according to electrostatic potential (red-negative, blue-positive). Molecule A is colored grey and displayed in ribbon representation with P-Thr612, Thr613, Thr614 and Ile620 shown as sticks. (**b**) Stereo view showing P-Thr612 from molecule A (grey) bound to the Pi-pocket of molecule B (cyan). Residues involved in interactions are shown as sticks and are labeled black for molecule A and blue for molecule B. Interactions are indicated by dashed lines. (**c**) The kinase activity of A-CAT-Δ809 was assayed in the presence of peptides with the sequences QQG(p)TMVMPD (closed circles) or a QQG(p)TMSMPD (open circles). Hyperbolic curves fit to the data yielded a K_d_ of 38 ± 12 μM and a V_max_ of 61 ± 4% for the QQG(p)TMVMPD peptide and a K_d_ of 98 ± 12 μM and a V_max_ of 51 ± 3% for the QQG(p)TMSMPD peptide. Kinase activities are reported as a percentage of wild-type A-CAT activity.

The free energy gained upon interface formation (Δ^i^G), calculated using PISA, suggests that the interfaces are likely to represent crystal contacts rather than to be of biological significance (Table S1)^37^. However, the oligomeric nature of MHCK-A, which brings multiple α-kinase domains into close proximity, should strongly favor the formation of intermolecular contacts. In this regard, it is interesting to note that the A-C interface buries the front surface of the C-lobe, including residues required for catalysis such as Phe720, Lys722 and Asp756, and thus is likely to block substrate phosphorylation (Fig. 2c). The interface between molecules A and D buries the top part of the N-lobe of molecule A and the lower part of the C-lobe of molecule D, including Tyr727, the N/D-loop and αE helix. Of most interest is the interface between molecules A and B (identical to the G-E interface), which involves the insertion of a phosphothreonine residue (P-Thr612) from molecule A into the Pi-pocket of molecule B (Fig. 3a). This interface provides the first example of a phosphopeptide ligand bound to the Pi-pocket.

### Structure of a phosphopeptide ligand bound to the Pi-pocket

Thr612 is located within a sequence unique to MHCK-A which is inserted between the β6 and β7 strands. The insert forms an extended loop that wraps around the rear part of the N-lobe (Fig. 2c). Thr612 is phosphorylated in all molecules in the Apo-A-CAT asymmetric unit, in agreement with mass spectrometry data that identifies it as an autophosphorylation site^27^. The A-B interface has the smallest buried surface area yet exhibits the greatest theoretical thermodynamic stability of any of the crystal interfaces (Supplementary Table 1). The binding of P-Thr612 to the Pi-pocket contributes an estimated −7.3 kcal/mol of the total Δ^i^G of −9.4 kcal/mol. The interactions formed by the P-Thr612 phosphoryl group are equivalent to those formed by Pi and involve hydrogen bonds with the side chains of Lys684, Arg734 and Thr736 and the main chain amide of Ser735 (Fig. 3b). The threonine side chain methyl group participates in hydrophobic interactions with the side chains of Pro683 and Val799 in molecule B, which may account for the specificity that the Pi pocket exhibits for phosphothreonine^29^. Thr614 and Ile620 in molecule A are involved in hydrophobic interactions with Ala798 and Lys684 in molecule B, respectively (Fig. 3a,b). To test whether a hydrophobic residue in the P + 2 position favors binding to the Pi-pocket, experiments were carried out using a truncated A-CAT (A-CAT-Δ809) that lacks the activating Thr825 site of autophosphorylation and two phosphopeptides: one with a valine and one with a serine at the P + 2 position (QQG(p)TM**V**MPD and QQG(p)TM**S**MPD) (Fig. 3c). Similar to results reported previously, the QQG(p)TMVMPD peptide restored ~60% of kinase activity with a K_d_ of 38 ± 12 μM^29^. The QQG(p)TMSMPD peptide was less effective, yielding a K_d_ of 98 ± 20 μM and a maximal kinase activity of ~50%. The results are consistent with the conclusion that a hydrophobic residue at the +2 position following the phosphothreonine enhances binding to the Pi-pocket.

The Pi-pockets of the other molecules in the tetramer (A, C and D) are filled by P-Thr612 residues in neighboring asymmetric units. In all cases the mode of binding to the Pi-pocket is identical to that for the A-B interface.

### Apo-A-CAT has an open but obstructed catalytic cleft

**Figure 4:**
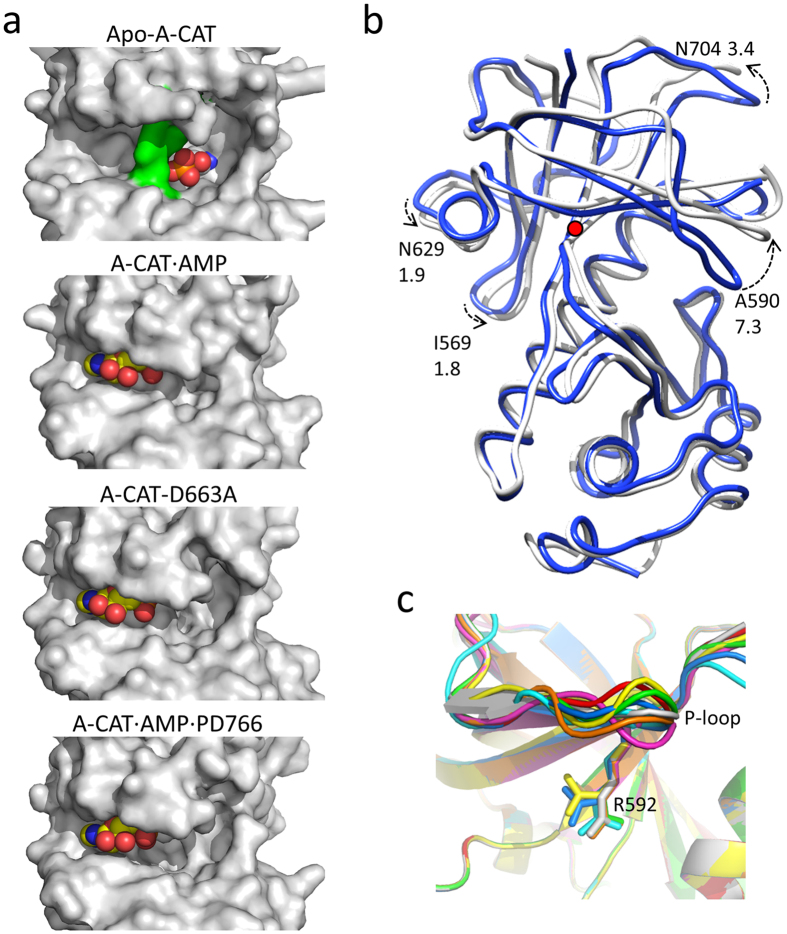
Rotation of the N-lobe opens up the catalytic cleft of Apo-A-CAT. (**a**) Surface representations of Apo-A-CAT, A-CAT·AMP, A-CAT-D663A and A-CAT·AMP·P-D766 showing the catalytic cleft. In the Apo-A-CAT structure P-Asp663 is shown as spheres and Arg592, Tyr647 and Asp766, which form a barrier dividing the cleft, are colored green. In the other structures AMP is shown as spheres. (**b**) Superimposition of the structures of Apo-A-CAT (white) and A-CAT-AMP (blue). Broken arrows with numbers show the direction and displacement (in Å) for the α-carbons of the indicated residues. The red dot indicates the pivot point for the N-lobe rotation (Trp692). (**c**) Superimposition of the eight molecules in the Apo-A-CAT asymmetric unit shows variability in the position of the P-loop and Arg592 (shown as sticks). Molecules are colored as in Fig. 2a.

Molecule D had the most residues well-defined in the electron density map and so was chosen as the representative Apo-A-CAT structure. Comparisons of molecule D with other molecules in the tetramer yielded RMSD values less than 1.2 Å. The catalytic cleft is more open in Apo-A-CAT than in nucleotide-bound structures and is divided in two by a barrier, which will be discussed in more detail below (Fig. 4a). The volumes of the left and right-hand sides of the Apo-A-CAT catalytic cleft, calculated using the CASTp program^38^, are 396 Å^2^ and 1314 Å^2^, respectively, giving a total volume for the cleft of 1710 Å^2^. For comparison, the closed inter-lobe cleft of A-CAT·AMP has a volume of 988 Å^2^.

**Figure 5:**
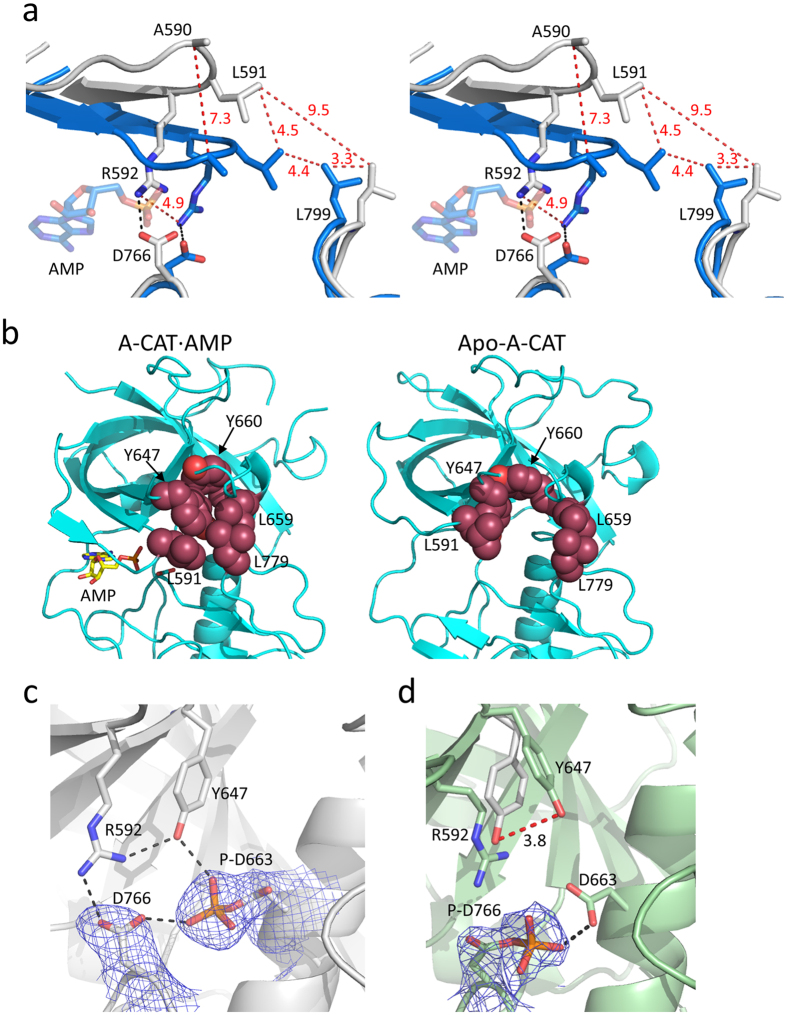
Phosphorylation of Asp663 alters the conformation of the A-CAT active site. (**a**) Stereo view showing the P-loop and N/D-loop of Apo-A-CAT (grey) and A-CAT·AMP (blue). In Apo-A-CAT the side chain of Arg592 occupies the same position as the α-phosphoryl group of AMP (shown as semi-transparent sticks) in A-CAT·AMP. Distances are given in Å for residues connected by red dashed lines. Black dashed lines indicate the interaction between Arg592 and Asp766. (**b**) Hydrophobic residues (Leu591, Tyr647, Leu659, Tyr660 and Leu779; shown as red spheres) assemble in A-CAT·AMP (left panel) to organize the active site but are disassembled in Apo-A-CAT (right panel). (**c**) Asp663 is phosphorylated in Apo-A-CAT. The purple mesh shows the 2F_o_−F_c_ electron density map contoured at the 2σ level. Interactions supporting Arg592 are indicated by black dashed lines. (**d**) The structure of A-CAT·AMP·PD766 indicates that Asp663 is hydrogen bonded to the phosphoryl group of P-Asp766. The purple mesh shows the 2F_o_−F_c_ electron density map contoured at the 2σ level. Tyr647 in Apo-A-CAT (white) is superimposed to illustrate the displacement of this residue (red dashed line) induced by Asp663 phosphorylation.

Superimposition of the Apo-A-CAT and A-CAT·AMP structures shows that there is a rotation of the N-lobe relative to the C-lobe (Fig. 4b). The pivot point for the rotation is Trp692 at the start of the β9 strand in the polypeptide chain that runs from the bottom of the αC helix to the N-lobe. The rotation lifts the P-loop upwards and forces the β1-loop-β3 element at the back of the N-lobe towards the central αC helix. Although most N-lobe residues are displaced by no more than 1.5–3.5 Å, Ala590 in the middle of the P-loop is shifted upwards a distance of 7.3 Å. In contrast, the highly conserved Arg592 at the C-terminal end of the P-loop moves upwards by only 3.3 Å. As a result, the P-loop is twisted as it moves upwards, causing the side chain of Arg592 to be thrust forwards into the middle of the catalytic cleft (Figs 4c and 5a). The P-loop is quite flexible in Apo-A-CAT, with an average B factor greater than 84 Å^2^, and takes up somewhat different conformations in molecules A to H in the asymmetric unit (Fig. 4c). In all cases, though, the Arg592 side chain is re-positioned towards the front, middle of the catalytic cleft, where it occupies the same space as the α-phosphoryl group of AMP (Fig. 5a). Thus, nucleotide binding to the Apo-A-CAT catalytic cleft is physically blocked.

The upwards movement of the β5 strand, P-loop and β6 strand serves to open up the catalytic cleft of Apo-A-CAT. In addition, the top part of the N/D-loop shifts outwards, so that the distance from Leu591 in the P-loop to Leu799 in the N/D-loop is increased from 4.4 Å in A-CAT·AMP to 9.5 Å in Apo-A-CAT (Fig. 5a). The conformational changes disrupt a grouping of five hydrophobic residues (Leu591 in the P-loop, Tyr647 in the β7 strand, Leu659 and Tyr660 at the start of the αC helix and Leu779 in the N/D-loop) that serves to organize and position the structural elements that comprise the right-hand, peptide-binding side of the catalytic cleft (Fig. 5b).

### Asp663 is phosphorylated in Apo-A-CAT

Asp766 is not phosphorylated in Apo-A-CAT. Instead, all eight molecules in the asymmetric unit exhibited a strong difference Fourier map that allows a phosphoryl group to be unambiguously placed in a covalent bond with the side chain of Asp663 (Fig. 5c). The P-Asp663 side chain projects outwards from the αC helix, with the phosphoryl group hydrogen bonded to the side chains of Asp766 and Tyr647. These interactions reorient the Tyr647 and Asp766 side chains so that they interact with Arg592 (Fig. 5c and 5d). The phosphorylation of Asp663 therefore helps to establish a network of interactions that reinforces the inhibitory position of Arg592 in the catalytic cleft.

In structures that contain a phosphorylated Asp766 residue, the phosphoryl group is hydrogen bonded to the side chain of Asp663 (Fig. 5c). This arrangement suggests the possibility that the phosphoryl group is directly transferred from P-Asp766 to Asp663.

### Crystal structure of the D663A mutant

To gain further insight into the function of Asp663, an A-CAT D663A mutant was crystallized and its structure was solved to a resolution of 2.5 Å. Although A-CAT-D663A was crystallized in the presence of MgATP, AMP was present in the catalytic cleft. Asp766 was not phosphorylated in the A-CAT-D663A structure.

**Figure 6:**
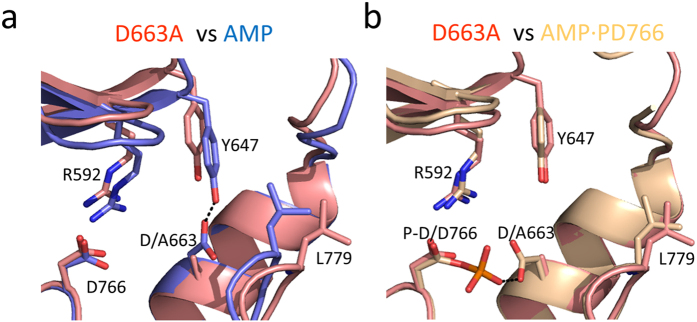
Effects of Asp663 and Tyr647 mutations on the conformation of the catalytic cleft and catalytic activity. The structures of A-CAT-D663A (salmon) and (**a**) A-CAT-AMP (blue) and (**b**) A-CAT·AMP·P-D766 (wheat) are superimposed. The D663A mutation and phosphorylation of Asp766 induce similar conformational changes within the right-hand side of the catalytic cleft.

A surface representation of A-CAT-D663A shows that the left-hand side of the catalytic cleft is closed whereas the right-hand side is open (Fig. 4a). The D663A mutation eliminates a hydrogen bond interaction between Asp663 and the Tyr647 residue located at the end of the β7 strand (Fig. 6a). The hydrogen bond with Asp663 attracts Tyr647 towards the C-lobe, so that it can interact with Leu659 in the αC helix, which in turn interacts with Leu779 in the N/D-loop. The absence of the hydrogen bond in A-CAT-D663A causes Tyr647 to move upwards by 1.8 Å (Fig. 6a). The upwards movement is transmitted to the adjacent β6 strand and P-loop and disrupts the hydrophobic interaction with Leu659, causing the side chains of Leu659 and Leu759 to moves outwards by 3.0 Å.

Interestingly, the right-hand side of the catalytic cleft is open in structures that have a phosphorylated Asp766 residue, such as A-CAT·AMP·P-D766 (Figs 4a and 6b). This can be attributed to the preference of Asp663 to hydrogen bond to the P-Asp766 phosphoryl group rather than Tyr647.

### Asp663 and Tyr647 play roles in protein phosphorylation

The mutation of Asp663 to alanine or serine increased the affinity of A-CAT for nucleotide, as measured by the binding of mant-ATP and mant-ADP (Table 2). The D663A and D663S mutants retained between 25 and 50% of wild-type ATPase activity, but exhibited extremely low kinase activity (~1% of wild-type). The results indicate that Asp663 specifically effects the kinase reaction, possibly by decreasing the binding of the protein substrate. Similarly, the mutation of Tyr647 to phenylalanine had little effect on nucleotide binding and inhibited kinase activity (~0.05% of wild-type) to a much greater extent than ATPase activity (~25% of wild-type) (Table 2). A Y647A mutation weakened the binding of mant-ADP and mant-ATP and significantly increased the K_m_ for ATP in the ATPase and kinase assays. The Y647A mutation reduced kinase activity to 4% and ATPase activity to 15% of wild-type A-CAT.

## Discussion

The X-ray crystal structure of Apo-A-CAT reveals a novel autoinhibited conformation for the α-kinase domain. The active site cleft is an open state, but nucleotide binding is blocked by the side chain of the conserved Arg592 residue in the P-loop. Unexpectedly, the structure contains a phosphorylated Asp663 residue, which may play a role in rearranging the active site in order to stabilize the autoinhibited conformation. In addition, Apo-A-CAT provides the first experimental evidence that the Pi-pocket allosteric site recognizes and binds phosphopeptide ligands.

The α-kinase catalytic cleft, located at the junction of the N- and C-lobes, must recognize both nucleotide and protein substrates. Adenine binds into the far left-hand portion of the catalytic cleft, with the ribose moiety and phosphoryl groups extending towards the middle of the cleft. Although no structure is available for an α-kinase in a complex with a peptide substrate, mutagenesis and modeling studies indicate that peptides are recognized by the right-hand side of the cleft and N/D-loop^27^,^28^. In all nucleotide-bound structures of A-CAT the left-hand side of the catalytic cleft is in a closed conformation with the P-loop clamped down over the adenine base, ribose moiety and phosphoryl groups (Fig. 4a). Because A-CAT-adenosine is in a closed state, it is clear that the phosphoryl groups are not required to hold the N-lobe in place. Moreover, A-CAT makes few contacts with the ribose moiety, so that the closed state must depend primarily on interactions with the adenine base. Hydrophobic interactions with the adenine base are made by Phe586 in the β5 strand and Val643 in the β7 strand. These two residues form part of a hydrophobic catalytic spine, comparable to that described in conventional protein kinases, that connects the N-lobe through the adenine base to the αD helix in the C-lobe (Supplemental Figure S1)^39^. The invariant Lys645 residue in the β7 strand is also involved in binding the nucleotide through formation of a hydrogen bond with the adenine N2 atom. The loss of interactions with the adenine base is likely to account for the movement of the N-lobe relative to the C-lobe in Apo-A-CAT, which greatly enlarges the catalytic cleft by lifting the β5 strand, P-loop and β6 strands upwards.

The right-hand peptide-binding side of the catalytic cleft can open and close independently of the left-hand side of the cleft. A closed conformation, in which the side chains of Leu591 in the P-loop and Leu779 in the N/D-loop are nearly in contact, is found in structures that contain an unphosphorylated Asp766 residue, as exemplified by A-CAT·AMP (PDBID 3LKM). An open conformation, in which Leu591 and Leu779 are farther apart, is present in Apo-A-CAT, A-CAT-D663A and in structures that contain a P-Asp766 residue, such as A-CAT·ADP (PDBID 3LMH). As described in detail below, the conformation of the right-hand side of the catalytic cleft is controlled by a hydrogen bond between Asp663 and Tyr647.

The open catalytic cleft of Apo-A-CAT is divided by a barrier that sterically blocks the binding of the nucleotide α-phosphoryl group (Figs 4a and 5a). The main component of the barrier is the side chain of Arg592, which is translocated towards the front of the catalytic cleft by the upwards twisting movement of the P-loop that occurs in Apo-A-CAT. The inhibitory position of Arg592 is reinforced by interactions with Asp766 and Tyr647, which in turn are positioned through interactions with the P-Asp663 phosphoryl group. The phosphorylation of Asp663 has not previously been observed in A-CAT, although multiple structures contain a P-Asp766 residue. It has been proposed that the phosphorylation of Asp766 represents an intermediate step in the catalytic reaction^27^. The side chain carboxylate of Asp663 is within 2.9 Å of the phosphoryl group of P-Asp766, so it is plausible that when a peptide substrate is not available the phosphoryl group is instead transferred from P-Asp766 to Asp663. It is unlikely that Asp663 can directly accept a phosphoryl group from the nucleotide, since the Asp663 side chain is 7–10 Å distant from the nucleotide β- and γ-phosphoryl groups^27^. We propose that the phosphorylation of Asp663 helps to lock A-CAT into a nucleotide-free, autoinhibited conformation.

The function of the highly conserved Arg592 residue in the α-kinase P-loop has remained obscure. Mutation of Arg592 to alanine increases the K_m_ for ATP by 3-fold and decreases k_cat_ by 6-fold, so Arg592 clearly has an important, albeit non-essential, role in catalysis. Our results indicate that Arg592 also plays a key role in switching the α-kinase to an “off” state. The possibility that this is a conserved function for Arg592 gains some support from structural studies on TRPM7^26^. The structure of the TRPM7 α-kinase domain apoenzyme shows that a barrier remarkably similar to that present in Apo-A-CAT is formed in the middle of the catalytic cleft from the side chains of Arg1622 (Arg592) and Asp1775 (Asp766) (Supplemental Figure S2). However, the barrier is present only in the A subunit of the TRPM7 dimer and is accompanied by a disordering of both the P-loop and N/D-loop. The glutamic acid residue (Glu1672) that replaces Asp663 in TRPM7 does not interact with the barrier.

Biochemical evidence for a conformational change in the α-kinase domain that alters ATP binding has been obtained for the Ca^2+^-calmodulin-activated eEF2K^40^. eEF2K binds ATP in the presence and absence of Ca^2+^-calmodulin but can be UV cross-linked to ATP only in the presence of Ca^2+^-calmodulin. It can be speculated that the failure of ATP to cross-link to the inactive eEF2K may be caused by a rearrangement of active site residues similar to that described here for Apo-A-CAT.

Further insight into the function of Asp663 was gained by solving the crystal structure of the A-CAT-D663A mutant. A-CAT-D663A retains the ability to hydrolyze ATP and ADP, showing that Asp663 is not essential for catalytic activity. The left-hand side of the A-CAT-D663A catalytic cleft is closed but, interestingly, the right-hand side is open. This result demonstrates that the hydrogen bond formed by Asp663 with Tyr647 is critical to maintain the right-hand side of the cleft in a closed state and, furthermore, provides an explanation for the finding that Asp766 phosphorylation opens up the right-hand side of the cleft^27^. Asp663 preferentially hydrogen bonds to P-Asp766 rather than to Tyr647, so that the phosphorylation of Asp766 provides a reversible mechanism to disrupt the Asp663-Tyr647 hydrogen bond and open up the right-hand side of the cleft.

Tyr647 interacts with four other hydrophobic residues to organize the key elements in the N- and C-lobes that form the active site (P-loop, β5 strand, αC helix and N/D-loop) (Fig. 5B). In this sense, the A-CAT hydrophobic group may be comparable to the regulatory hydrophobic spine in conventional protein kinases that can be dynamically assembled or disassembled to control activity^39^. The re-orientation of the Tyr647 side chain in Apo-A-CAT disassembles the hydrophobic group and opens up the right-hand side of the active site. Interestingly, a Y647F mutation potently inhibited kinase activity but did not interfere with nucleotide binding, suggesting that it may stabilize a conformation of A-CAT that is unable to bind or properly orient the protein substrate. A Y647A mutation inhibited nucleotide binding, as well as ATPase and kinase activity. It can be speculated that the replacement of the bulky Tyr647 with the smaller alanine residue might increase the flexibility of the P-loop and β7 strand, which would be reflected in a decreased binding affinity for nucleotide.

The discovery of an autoinhibited conformation for A-CAT demonstrates that α-kinases, like conventional protein kinases, are dynamic enzymes that can switch between distinct active and inactive conformations^41^. If the bipartite conformation of the catalytic cleft in the autoinhibited state is a unique feature of α-kinases, it will provide an opportunity to develop highly specific small molecule inhibitors for this class of protein kinases. An important question that remains to be answered concerns the nature of the input signals that induce A-CAT to switch between the inactive and active conformations. In particular, further studies are needed to gain a better understanding of the conditions under which Asp663 is phosphorylated and dephosphorylated.

The eight molecules in the Apo-A-CAT asymmetric unit interact via a number of different interfaces. None of the interfaces are predicted to be stable in solution, but it is possible that in MHCK-A, where multiple α-domains cluster together at one end of a rod-like coiled-coil domain, they help to organize and/or regulate the α-kinase domains^16^. Of most interest is the finding that the Pi-pocket allosteric site is occupied by a phosphothreonine residue (P-Thr612) from a neighboring molecule. The Apo-A-CAT structure is the first in which the Pi-pocket is bound to a phosphopeptide and provides evidence that the Pi-pocket can mediate intermolecular interactions.

A-CAT and eEF2K are activated by the autophosphorylation of a conserved threonine in the C-terminal linker (Thr825 in A-CAT; Thr348 in eEF2K), which is then proposed to act as an intramolecular ligand for the Pi-pocket^28^,^29^,^30^. A crystal structure of A-CAT with P-Thr825 bound to the Pi-pocket has not, however, been obtained and in Apo-A-CAT it is P-Thr612, rather than P-Thr825, that occupies the Pi-pocket. The Apo-A-CAT structure shows that the residue in the P + 2 position (Thr614) participates in hydrophobic interactions with the Pi-pocket. Experiments using synthetic phosphopeptides confirmed that a hydrophobic residue at the P + 2 position enhanced binding to the Pi-pocket, which is consistent with the sequences of the A-CAT Thr825 ((p)TMV) and eEF2K Thr348 ((p)TIL) autophosphorylation sites. The Pi-pocket may therefore be a relatively low-specificity site, and the binding of P-Thr612 rather than P-Thr825 to the Pi-pocket may simply reflect the manner in which the Apo-A-CAT molecules are packed into the crystal structure. It is also likely that the high protein concentration present in the crystal allows P-Thr612 to efficiently compete for binding to the Pi-pocket. On the other hand, the low concentrations of A-CAT and eEF2K that are employed in solution kinase assays are likely to strongly favor an intramolecular interaction with P-Thr825/P-Thr348.

## Materials and Methods

### Crystallization, data collection and processing

A-CAT and the A-CAT-D663A mutant were expressed in *E. coli*, purified and concentrated to a concentration of 8 mg/mL as described^27^. Crystals of A-CAT in the unliganded state (Apo-A-CAT) were obtained using the hanging-drop vapor-diffusion method. The crystallization buffer consisted of 5% PEG (w/v) 8000, 20% (w/v) PEG 300, 10% glycerol and 0.1 M Tris-HCl, pH 9.0. Large, fluffy crystals which diffracted to a 4.5 Å resolution were obtained after one week at 4 °C. Through a divalent ion screen, it was found that 0.1 M MgCl_2_ yielded higher quality crystals that diffracted to 2.9 Å. The A-CAT-D663A mutant solution contained 5 mM MgCl_2_ and 1.5 mM ATP. Crystallization drops were set up by mixing 2 μl of the solution containing A-CAT-D663A, 2 μl of mother liquor containing 0.15 M NaH_2_PO_4_, 20% (w/v) PEG 8000 and 0.1 M Tris-HCl, pH 8.0 and 0.6 μl of 2.5% dichloromethane. The crystallization plates were set up at 4 °C, and then moved into 20 °C after ~50 hours. Crystals that diffracted to 2.5 Å were obtained after one week. The crystals were cryoprotected with 25% ethylene glycol mixed with the precipitant solution and were then flash-cooled in liquid nitrogen. X-ray diffraction data were collected on the 23-ID-B and 23-ID-D beam lines at the Argonne National Laboratory (APS). Data for the apo A-CAT structure were processed using HKL2000^42^ and data for the A-CAT-D663A structure were processed using XDS^43^.

### Structural Determination and Refinement

Crystal structures were determined by the molecular replacement method using the CCP4 suit program PHASER^44^. The previously determined monomer structure of A-CAT (PDB ID: 3LKM), devoid of AMP, Zn^2+^ and Mg^2+^ions, was used as the search model. For the Apo-A-CAT data, it was indexed to belong to orthorhombic space group and P2(1)2(1)2(1) gave the highest likelihood Gain and Z scores from the model search. The obtained model was refined with CNS^45^. However, the R factor and free R factor remained above 40% and 48%, respectively. Use of the phenix.xtriage program showed that the data had a significant off-origin peak of 48.75% that indicated pseudo-translational symmetry in the crystal and twinning fraction of 46.7% for h, -k, -l^46^. After the data were re-indexed from H K L to K H -L with monoclinic space group P2(1), molecular replacement gave a clear solution. Refinement was carried out using PHENIX^47^ and REFMAC5 with twinning refinement^48^. Manual model rebuilding was performed with COOT^49^. The C-terminus of Apo-A-CAT (residues 819–841) had poor electron density in the crystal structure and this segment could not be built. The A-CAT-D663A structure was determined by direct refinement against the crystal structure of A-CAT in complex with AMPPCP (PDB ID: 3LLA), devoid of ligands using PHENIX^47^. Unambiguous density was present in the active site for AMP. The structure was built by iterative cycles of manual building in COOT^49^ followed by refinement in PHENIX^47^. MolProbity was used for structural validation, including Ramachandran plot analysis^50^. Structural refinement statistics are summarized in Table 1.

### Kinase and ATPase Assays

Assays were carried out at 22 °C in a buffer containing 2 mM MgCl_2_, 1 mM dithiothreitol and 20 mM TES, pH 7.0. Kinase assays contained 20 μM myelin basic protein (MBP), 0.5–2 μM A-CAT and 200 μM [γ-^32^P]ATP (specific activity 100 cpm/pmol). At each time point, 20 μl aliquots of the reaction mixture were spotted onto squares of Whatman P81 phosphocellulose paper, which were then washed 1% phosphoric acid, immersed in ScintiVerse Universal LS Mixture (Fisher Scientific), and counted using a Beckman LS 9000 scintillation counter. Assays to measure A-CAT-Δ809 activity in the presence of the QQG(p)TMVMPD and QQG(p)TMSMPD peptides were carried out as described^29^. ATPase activity was assayed using 250 μM ATP and release of inorganic phosphate (Pi) was measured using the Pi ColorLock Gold phosphate detection system (Innova Biosciences). Data from the initial linear portion of the time courses were used to calculate kinase and ATPase activity. Nucleotide binding assays using mant-ATP and mant-ADP were carried out as previously described^51^. Results represent the mean and standard deviation of at least 3 separate experiments.

## Additional Information

Accession codes: The Apo-A-CAT and A-CAT-D663A crystal structures have been deposited in the Protein Data Bank under accession codes 5E4H and 5DYJ, respectively.

**How to cite this article**: Ye, Q. *et al*. Structure of the *Dictyostelium* Myosin-II Heavy Chain Kinase A (MHCK-A) α-kinase domain apoenzyme reveals a novel autoinhibited conformation. *Sci. Rep*. **6**, 26634; doi: 10.1038/srep26634 (2016).

## Supplementary Material

Supplementary Information

## Figures and Tables

**Table 1:** Crystallographic statistics.

Data Set	Nucleotide-free Apo	D663A Mutant
Data-collection statistics		
X-ray source	Beam-line 23ID-B, APS	Beam-line 23ID-B, APS
Wavelength (Å)°	1.0332	1.0332
Space group	*P*2_1_	*P*2_1_2_1_2
Unit Cell parameters	*a* = 83.298	*a* = 84.14
(Å, °)°	*b* = 103.469	*b* = 110.25
	*c* = 167.811	*c* = 79.25
	*α = γ* = 90, *β* = 89.99	*α = β = γ* = 90
No. of molecules in asymmetric unit	8	2
Solvent content (%)	52.4	54.5
Resolution range (Å)	40–2.9 (3.0–2.9)	20–2.5 (2.7–2.5)
No of reflections	405542	372289
No of Unique reflections	62233	26125
Redundancy	6.5(3.2)	14.3(14.7)
<*I/σ (I)* >	16.72 (1.34)	16.74(5.12)
Completeness (%)	98.3 (86.4)	99.7 (100.0)
*R*_merge_ (%)^a^	14 (62)	12.2 (63.3)
Refinement statistics		
Resolution (Å)	2.9	2.5
No. of reflections used in refinement	58572	26106
*R*_*cryst*_*./R*_*free*_^b^	0.241/0.267	0.191/0.231
R.m.s.d. bond length (Å)	0.012	0.002
R.m.s.d. bond angle (°)	1.581	1.326
No. of atoms		
Protein	15998	4041
PO4 ion	–	10
Zn ion	8	2
AMP	–	46
Water	64	101
Wilson B-factor (Å^2^)	75.0	42.28
Average B factor (Å2)		
Protein	80.12	47.97
PO4 ion	–	46.78
Zn ion	74.28	41.63
Nucleotide	–	51.58
Water	54.19	46.14
Ramachandran plot statistics		
Most favorable (%)	97.26	96.03
Allowed (%)	2.74	3.97
Disallowed (%)	0.00	0.00
PDB ID	5E4H	5DYJ

*R*_free_ is the cross-validation *R* factor for the test set (5%) of reflections omitted in model refinement.

^a^*R*_merge_ = |*I*_obs_ − <*I*>|/*I*_obs_, where *I*_obs_ is the intensity measurement and <*I*> is the mean intensity for multiply recorded reflections.

^b^*R*_work_ and *R*_free_ = |*F*_obs_ − *F*_calc_|/|*F*_obs_| for reflections in the working and test sets, respectively.

**Table 2:** Effect of Asp663 and Tyr647 mutations on A-CAT hydrolytic and kinase activities.

A-CAT	mant-ATP	mant-ADP	ATPase Activity		Kinase Activity	
	K_d_ (μM)	K_d_ (μM)	k_cat_ (*min*^*−1*^)	K_m, ATP_(*μM*)	k_cat_ (*min*^*−1*^)	K_m, ATP_(*μM*)
WT	4.8 ± 0.6	15 ± 2	1.9 ± 0.1	35 ± 4	5.8 ± 0.4	23 ± 5
D663A	3.2 ± 0.9	8 ± 2	1.0 ± 0.2	21 ± 5	0.063 ± 0.007	54 ± 25
D663S	2.1 ± 0.5	12 ± 2	0.44 ± 0.16	31 ± 7	0.062 ± 0.007	29 ± 16
Y647F	4.3 ± 1.0	15 ± 2	0.38 ± 0.07	64 ± 2	0.027 ± 0.007	65 ± 35
Y647A	10 ± 3	30 ± 2	0.29 ± 0.07	>500	0.22 ± 0.02	162 ± 38
