# Supplementary material for: Structure of the Dictyostelium Myosin-II Heavy Chain Kinase A (MHCK-A) α-kinase domain apoenzyme reveals a novel autoinhibited conformation
Source: Sci Rep. 2016 May 23;6:26634. doi: 10.1038/srep26634 (PMC4876393; doi:10.1038/srep26634)
Supplement: Supplementary Information [file srep26634-s1.pdf]

Structure of the *Dictyostelium* Myosin-II Heavy Chain Kinase A (MHCK-A) α-kinase domain apoenzyme reveals a novel autoinhibited conformation

Qilu Ye#, Yidai Yang#, Laura van Staalduinen, Scott William Crawley, Linda Liu, Stephanie Brennan, Graham P. Côté* and Zongchao Jia*

Department of Biomedical and Molecular Sciences, Queen's University, Kingston, ON, K7L 3N6, Canada

# co-first authors

*co-corresponding authors (coteg@queensu.ca; jia@queensu.ca)

Supplementary Information

Table S1. Analysis of the interfaces for molecules A-D in the Apo-A-CAT crystal structure using PISA [1].

Interface[1]	1 iN_{res}[2]	2 iN_{res}[2]	BSAS[3] (Å^2)	ΔiG[4] kcal/mol	ΔiG[5] P-value
**A-G**	20	20	595.8	3.7	0.910
**A-B**	8	10	291.2	-9.4	0.012
**A-C**	15	21	572.4	-3.5	0.511
**A-D**	15	12	373.6	-2.9	0.487
**B-D**	22	16	592.2	-4.3	0.508

[1] The two interacting monomers are labeled as in Fig. 2B.

[2] The number of interface residues for each interacting molecule.

[3] The buried solvent accessible surface (BSAS) is calculated as the difference in total accessible surface areas of isolated and interfacing structures divided by two.

[4] Indicates the solvation free energy gain upon formation of the interface, calculated as the difference in total solvation energies of isolated and interfacing structures.

[5] Measures the probability of getting a lower than obtained ΔiG if interface atoms equal to the observed interface area are picked randomly from the protein surface. A $P<0.5$ indicates interfaces with higher than average hydrophobicity, implying that the interface surface can be interaction-specific.

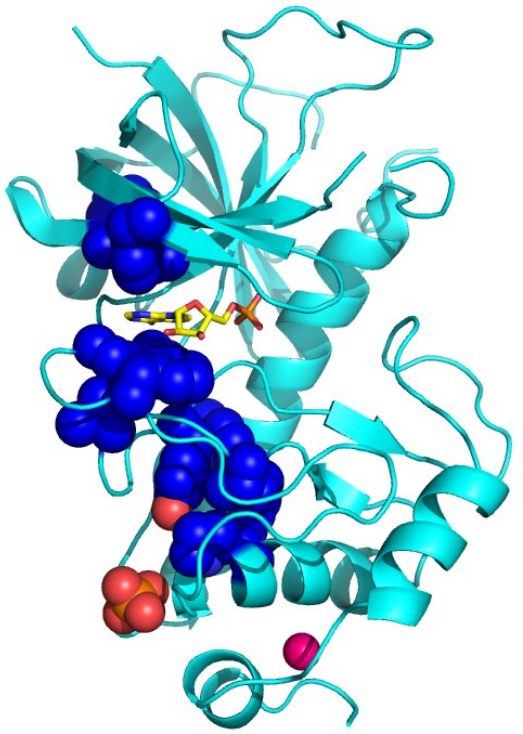

Figure S1. Catalytic spine in A-CAT·AMP. The catalytic spine is composed of hydrophobic residues that connect the N-lobe through the adenine base of the nucleotide to the αD helix in the C-lobe. Residues that comprise the hydrophobic spine are colored blue and shown as spheres. These residues are F586 and V643 in the N-lobe, F688, L689, L716, F720, I757, V760 and Y764 in loops in the C-lobe, and P737 and F740 in the αD helix. The Pi molecule in the Pi-pocket and the zinc atom (pink) are also shown as spheres.

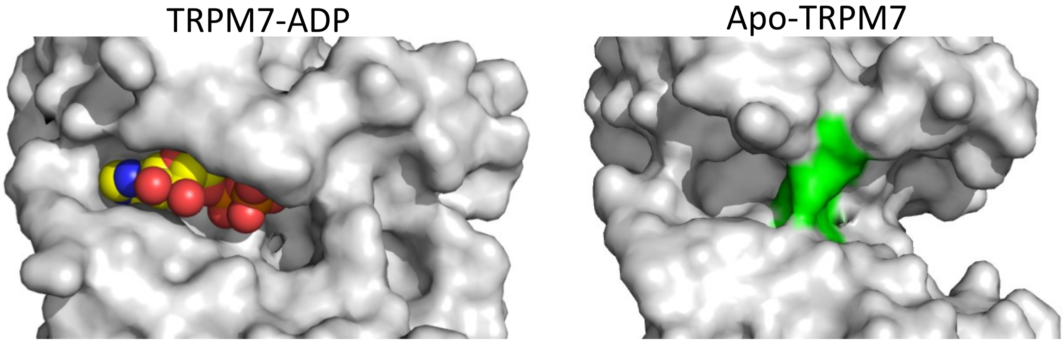

Figure S2. Catalytic cleft of the TRPM7 apoenzyme and ADP complex. Surface views of the α-kinase domain of TRPM7 bound to ADP (TRPM-ADP; PDB 1IAH) and in the absence of ligands (Apo-TRPM7; PDB 1IAJ). Arg1622 (Arg592) and Asp1775 (Asp766) are colored green to highlight the barrier in the middle of the Apo-TRPM7 catalytic cleft. Note that most of the P-loop and N/D-loop are disordered in Apo-TRPM7.

References

1. Krissinel, E. and Henrick, K. (2007) Inference of macromolecular assemblies from crystalline state. *J. Mol. Biol. 372*, 774-797.
